# Supplementary material for: Single sample pathway analysis in metabolomics: performance evaluation and application
Source: BMC Bioinformatics. 2022 Nov 14;23:481. doi: 10.1186/s12859-022-05005-1 (PMC9664704; doi:10.1186/s12859-022-05005-1)
Supplement: Supplementary file 1 — Additional file 1: Table S1. Method implementation details. Fig. S1. Overview of novel methods ssClustPA and kPCA for a single pathway example using simulated data based on the COVID dataset. Fig. S2. Performance of ssPA methods using the full pathway set (including redundancy) based on 3 randomly enriched pathways, derived using semi-synthetic data based on the IBD dataset. Fig. S3. Performance of ssPA methods using the full KEGG human pathway set (including redundancy) based on 3 randomly enriched pathways, derived using semi-synthetic data based on the COVID dataset. Fig. S4. ssPA method ability to rank highly the 3 randomly enriched pathways using the full set of Reactome pathways. Fig. S5. ssPA method performance in response to varying levels of pathway coverage. Fig. S6. ssPA method performance in response to varying levels of pathway coverage. Fig. S7. PCA scatter plots and density plots of PC1 scores obtained using the IBD data at the metabolite (upper panels) and pathway level using kPCA (lower panels). Fig. S8. Clustered heatmap of IBD data transformed to pathway scores using the kPCA method. Fig. S9. Pathway clusters derived using hierarchical clustering on COVID dataset transformed to pathway scores using kPCA (top 30 pathways). Table S2. Runtimes of ssPA methods, alongside GSEA* for comparison to conventional PA methods (average across 10 iterations). Table S3. Over-representation analysis results from IBD dataset. Table S4. Top 50 features in random forest model based on IBD data. [file 12859_2022_5005_MOESM1_ESM.docx]

**Single sample pathway analysis in metabolomics: performance evaluation and application**

Cecilia Wieder^1^, Rachel PJ Lai^2^, Timothy MD Ebbels^1^*

^1^ Section of Bioinformatics, Division of Systems Medicine, Department of Metabolism, Digestion, and Reproduction, Faculty of Medicine, Imperial College London, London, United Kingdom

^2^ Department of Infectious Disease, Faculty of Medicine, Imperial College London, London, United Kingdom

Corresponding author email: [t.ebbels@imperial.ac.uk](mailto:t.ebbels@imperial.ac.uk)

**Additional file 1: Supplementary information**

***Table S1: Method implementation details.*** *Methods are classified into categories based on the underlying algorithm used for scoring. Conventional pathway analysis methods i.e. non-single sample are denoted as “Conventional”.*

| Method | Implementation | Original publication | Method type |
| --- | --- | --- | --- |
| SVD | py-ssPA | Tomfohr, Lu, and Kepler  (2005) | DR |
| ssGSEA | GSVA (R) (Hänzelmann,  Castelo, and Guinney 2013) | Barbie et al. (2009) | GSEA |
| GSVA | GSVA (R) (Hänzelmann,  Castelo, and Guinney 2013) | Hänzelmann, Castelo, and  Guinney (2013) | GSEA |
| z-score | py-ssPA | Lee et al. (2008) | z-score |
| ssClustPA | py-ssPA | Present work | Clustering |
| kPCA | py-ssPA | Present work | DR |
| ORA | py-ssPA | Drâghici et al. (2003) | Conventional |
| GSEA | fGSEA (R) (Korotkevich, Sukhov, and Sergushichev  2019) | Subramanian et al. (2005) | Conventional |


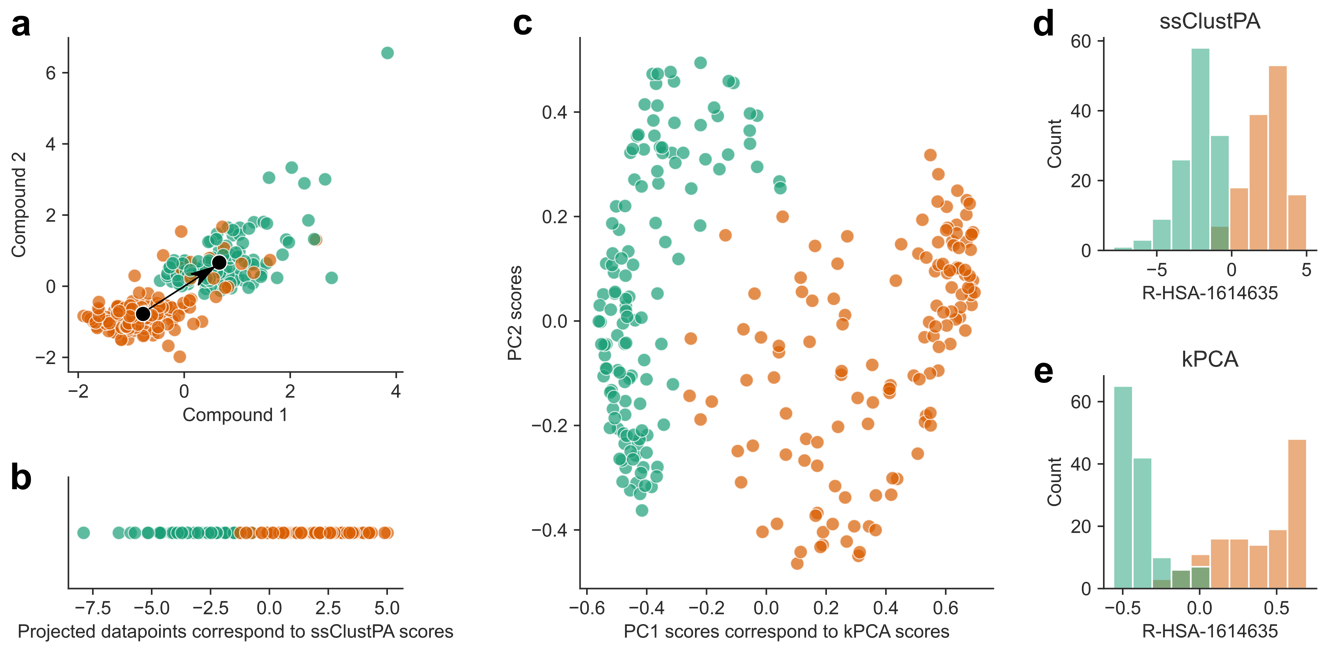


***Fig S1: Overview of novel methods ssClustPA and kPCA for a single pathway example using simulated data based on the COVID dataset****. Different coloured points are used to distinguish between case and control groups. a) ssClustPA overview. Scatterplot shows datapoints from two dimensions (metabolites) of the pathway matrix* $Z_{R-HSA-1614635}$ *containing all samples but only those metabolites present in R-HSA-1614635. Black dots represent cluster centroids identified by k-means. Scores are calculated by computing the unit vector between the two centroids (black arrow) and projecting this onto the original datapoints. b) ssClustPA scores. Datapoints correspond to those in a), shown after projection onto the unit vector between the two cluster centroids. c) kPCA overview. Scatterplot of kPCA scores (x-axis = PC1 and y-axis = PC2). PC1 scores correspond directly to pathway scores. d, e) Distribution of pathway scores across samples calculated using ssClustPA and kPCA respectively for pathway R-HSA-1614635.*


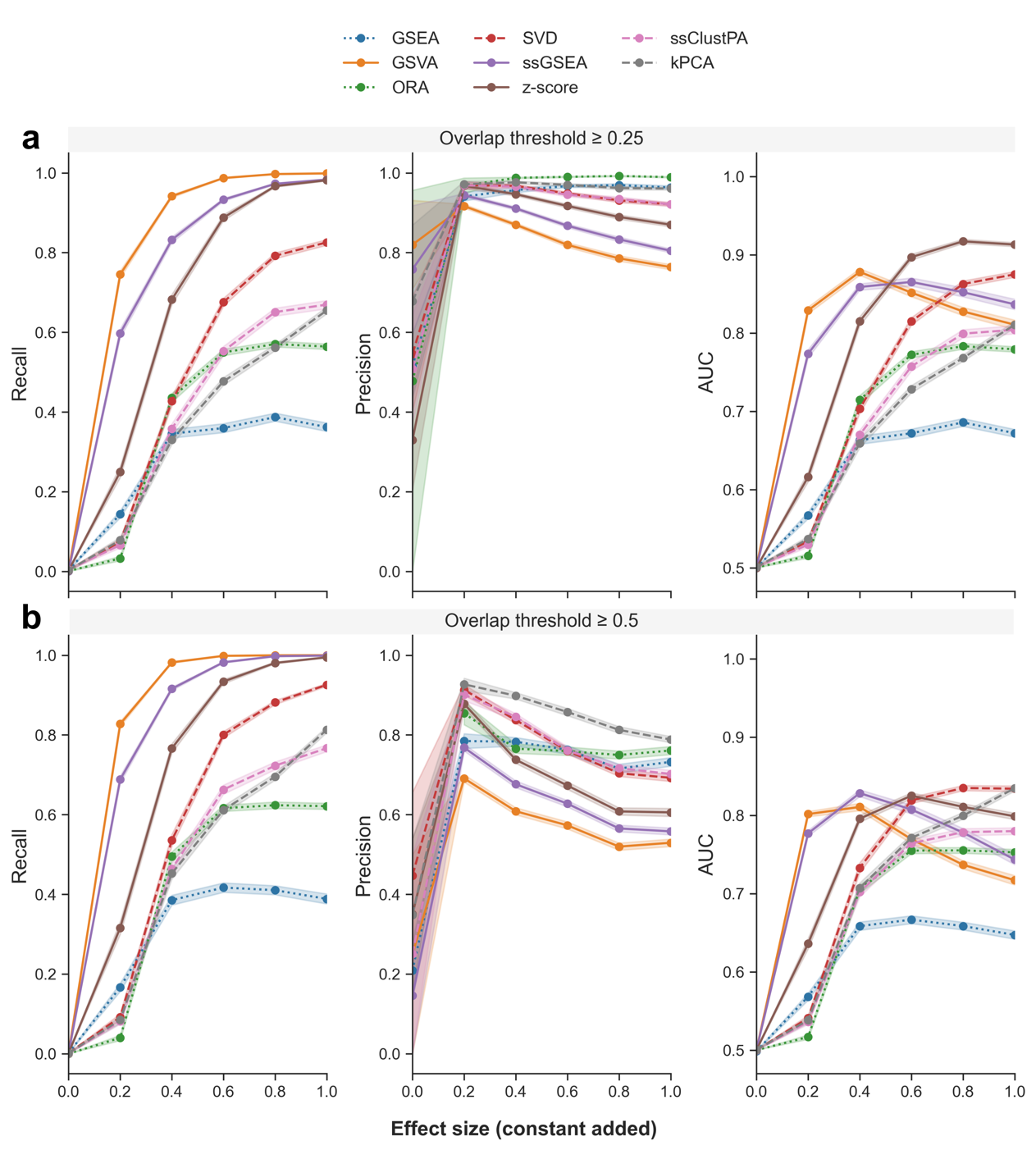


***Fig S2: Performance of ssPA methods using the full pathway set (including redundancy) based on 3 randomly enriched pathways, derived using semi-synthetic data based on the IBD dataset****. (a) top panel OC* $\geq$ *0.25, (b) bottom panel OC* $\geq$ *0.5. All metabolites in enriched pathways have identical effect size. Points show performance metrics averaged across 200 iterations. Shaded intervals represent average SEM. Dotted lines represent conventional pathway analysis methods, and dashed lines represent clustering/dimensionality-reduction based methods.*

*
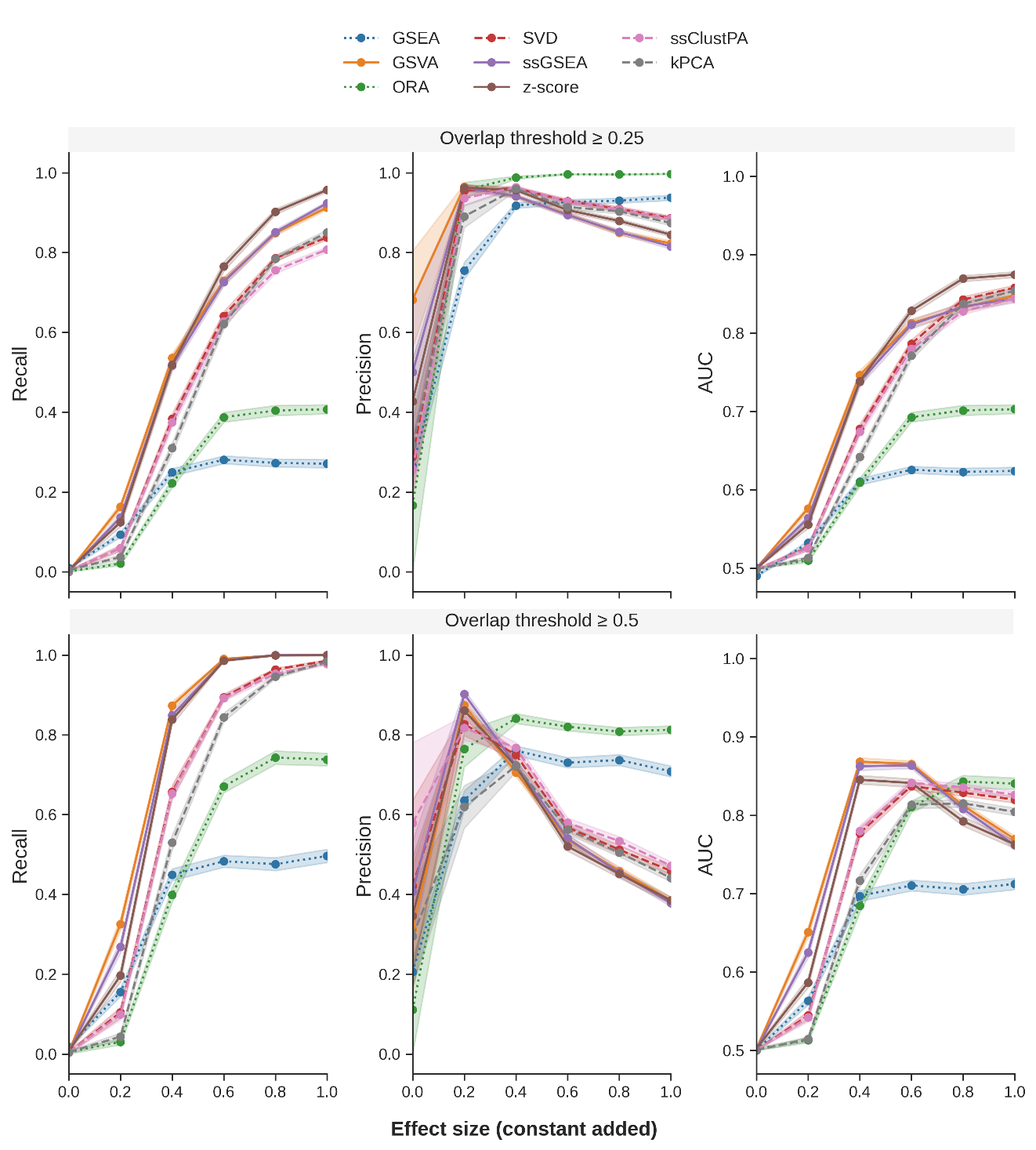
*

***Fig S3: Performance of ssPA methods using the full KEGG human pathway set (including redundancy) based on 3 randomly enriched pathways, derived using semi-synthetic data based on the COVID dataset****. (a) top panel OC* $\geq$ *0.25, (b) bottom panel OC* $\geq$ *0.5. All metabolites in enriched pathways have identical effect size. Points show performance metrics averaged across 200 iterations. Shaded intervals represent average SEM. Dotted lines represent conventional pathway analysis methods, and dashed lines represent clustering/dimensionality-reduction based methods.*


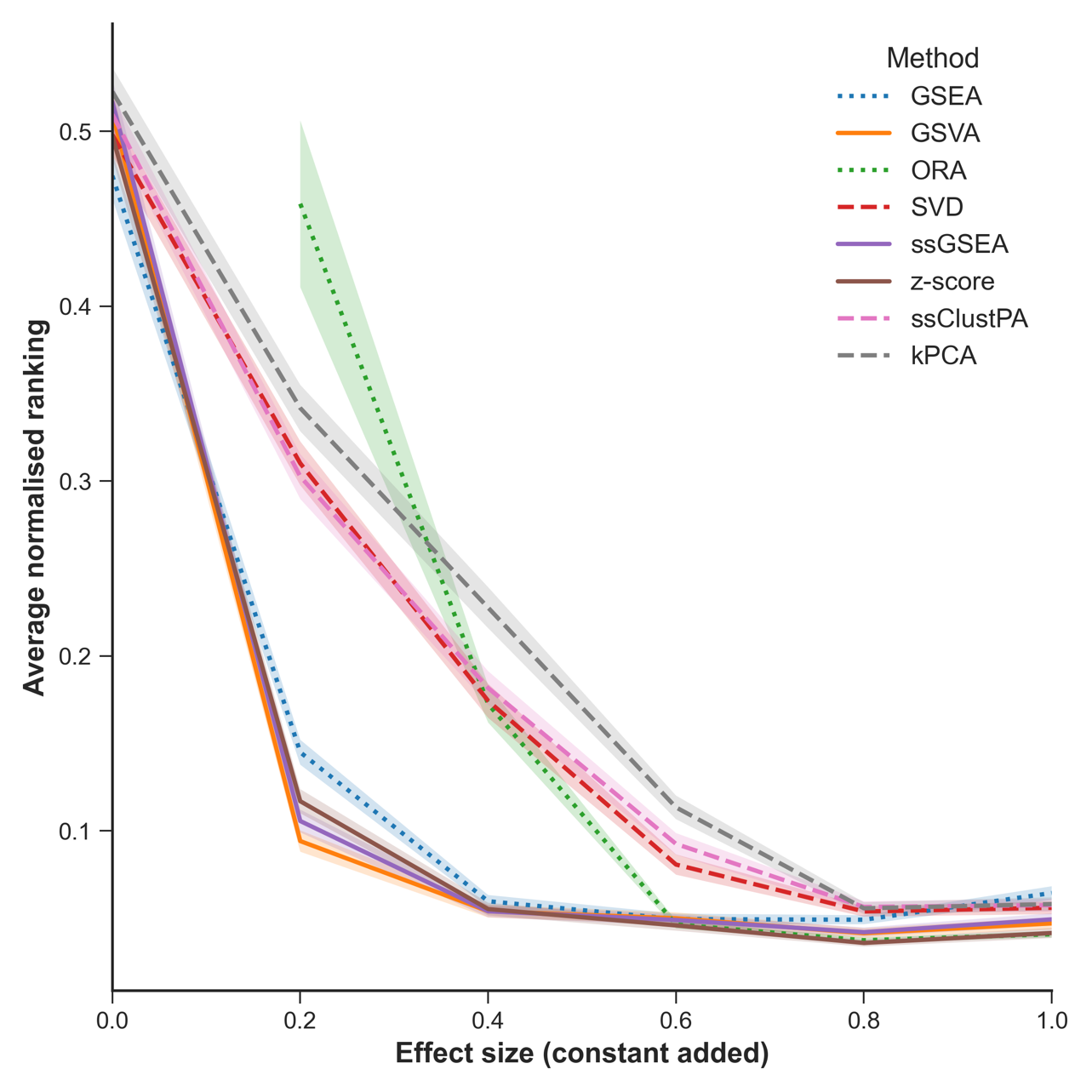


***Fig S4: ssPA method ability to rank highly the 3 randomly enriched pathways using the full set of Reactome pathways****. Average normalised ranking of 3 randomly selected enriched pathways shown on the y-axis. Shaded intervals represent standard error on the mean averaged over 200 iterations. Dotted lines represent conventional pathway analysis methods, and dashed lines represent clustering/dimensionality-reduction based methods.*

*
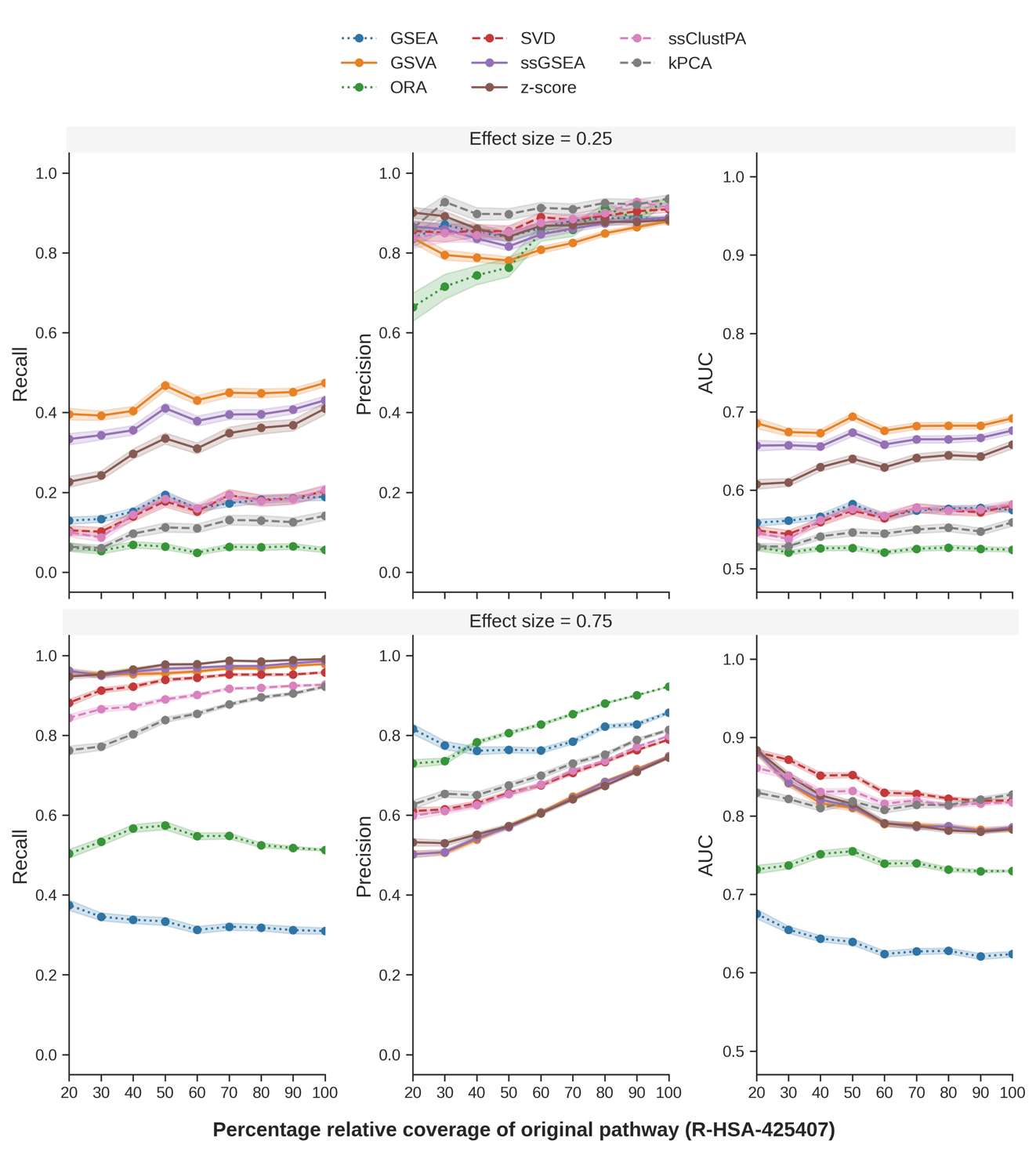
*

***Figure S5:*** ***ssPA method performance in response to varying levels of pathway coverage.*** *Pathway ‘R-HSA-425407’ (SLC-mediated transmembrane transport) had original coverage of 39 metabolites using the COVID dataset. We randomly deleted x% of the metabolites in this pathway from the dataset and calculated performance metrics at effect sizes of 0.25 and 0.75 with a fixed overlap coefficient of 0.5 to determine true positive pathways. Points show performance metrics averaged across 200 iterations. Shaded intervals represent average SEM. Dotted lines represent conventional pathway analysis methods, and dashed lines represent clustering/dimensionality-reduction based methods.*


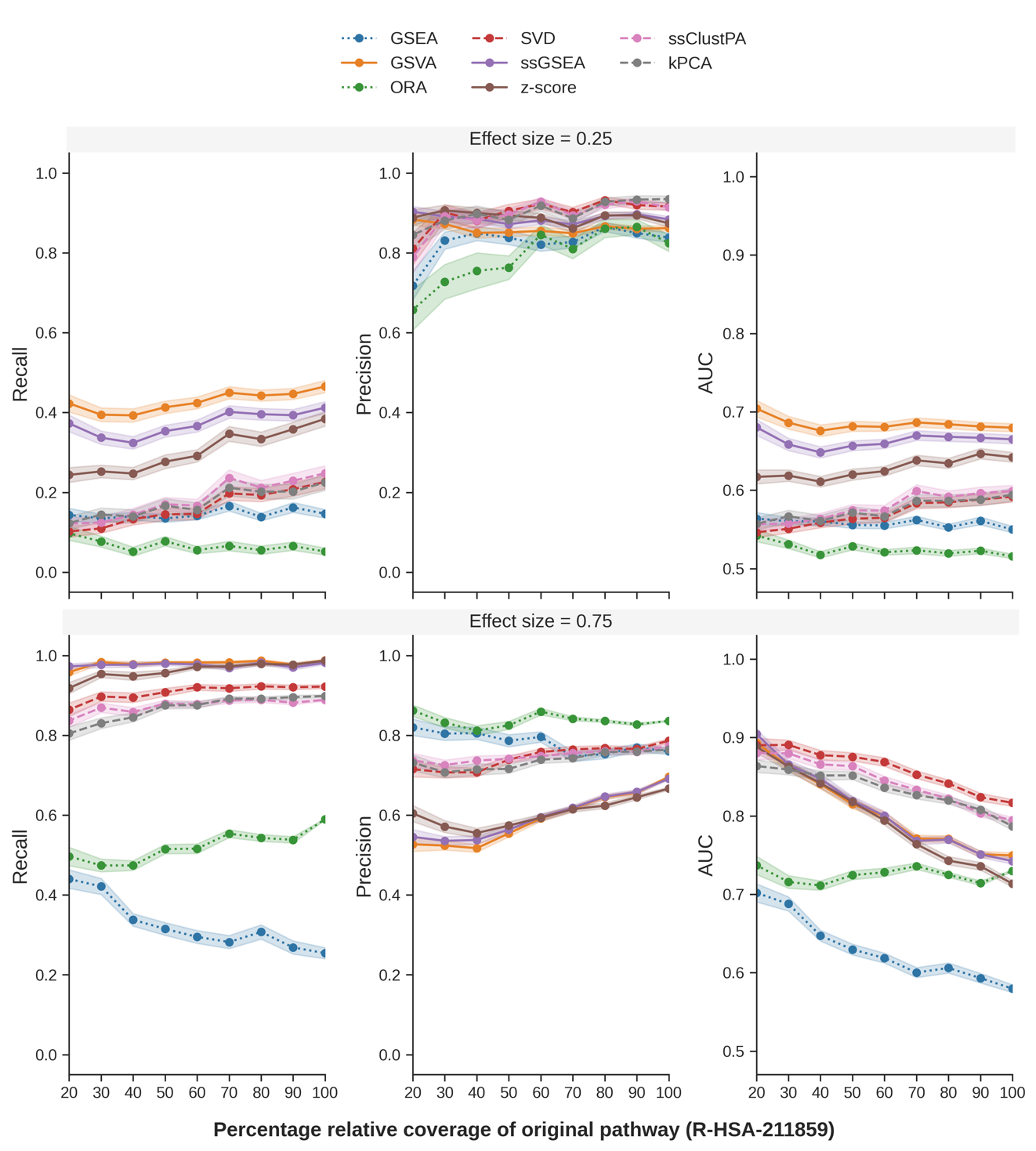


***Figure S6:*** ***ssPA method performance in response to varying levels of pathway coverage.*** *Pathway ‘R-HSA-211859’ (Biological oxidations) had original coverage of 26 metabolites using the COVID dataset. We randomly deleted x% of the metabolites in this pathway from the dataset and calculated performance metrics at effect sizes of 0.25 and 0.75 with a fixed overlap coefficient of 0.5 to determine true positive pathways. Points show performance metrics averaged across 200 iterations. Shaded intervals represent average SEM. Dotted lines represent conventional pathway analysis methods, and dashed lines represent clustering/dimensionality-reduction based methods.*


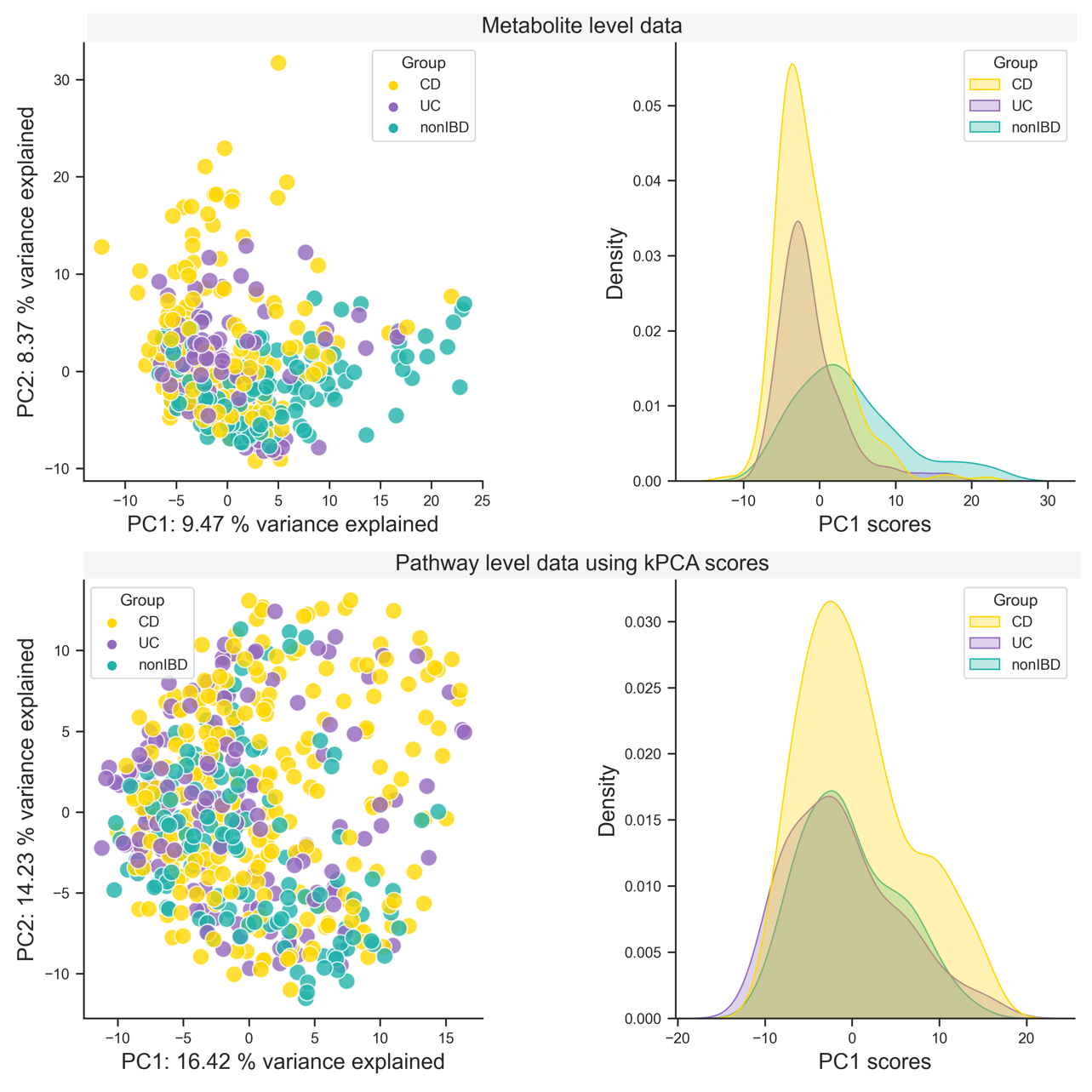


***Fig S7: PCA scatter plots and density plots of PC1 scores obtained using the IBD data at the metabolite (upper panels) and pathway level using kPCA (lower panels).***


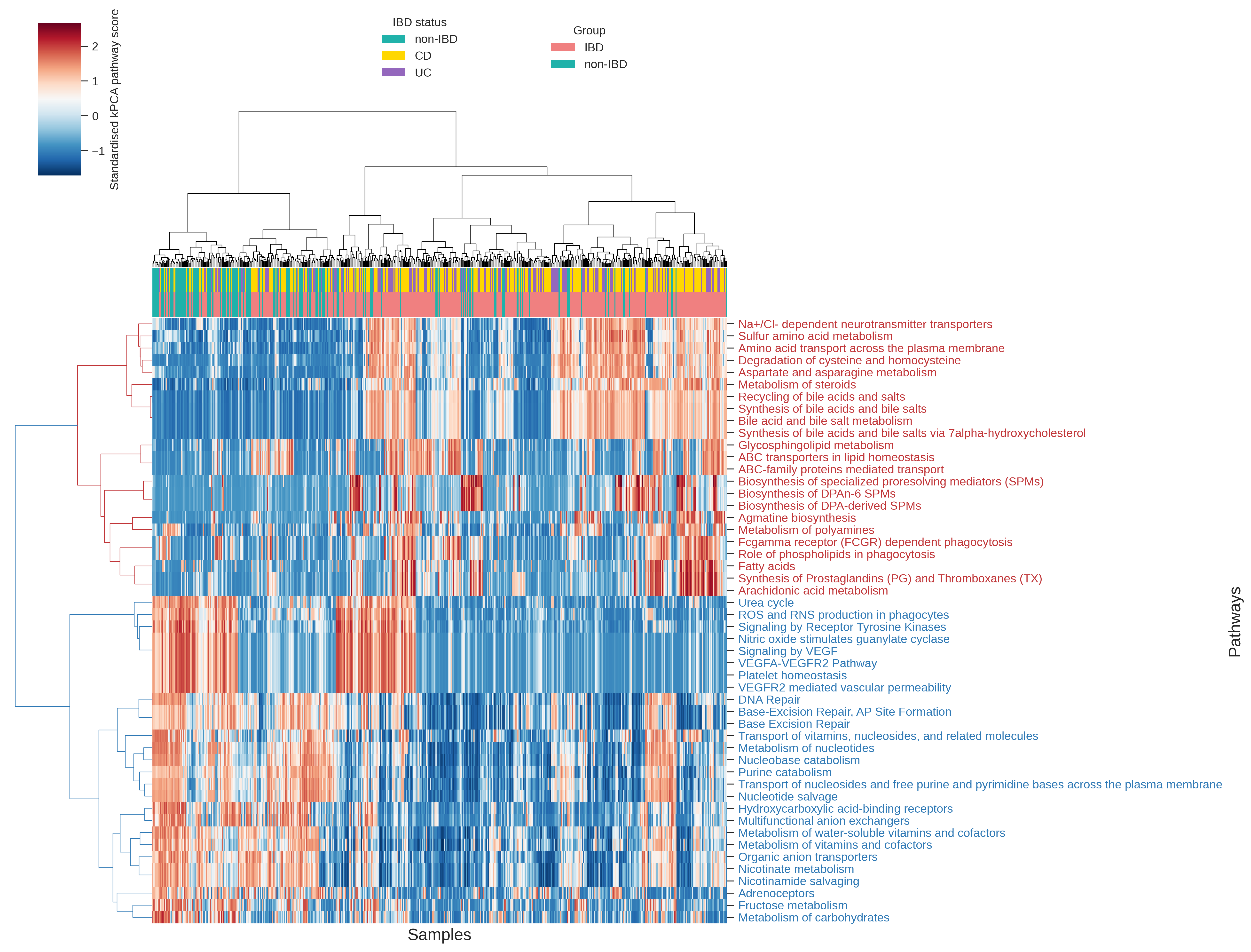


***Fig S8: Clustered heatmap of IBD data transformed to pathway scores using the kPCA method****. Top 50 pathways are used for clustering (performed on Euclidean distances using Ward linkage).*


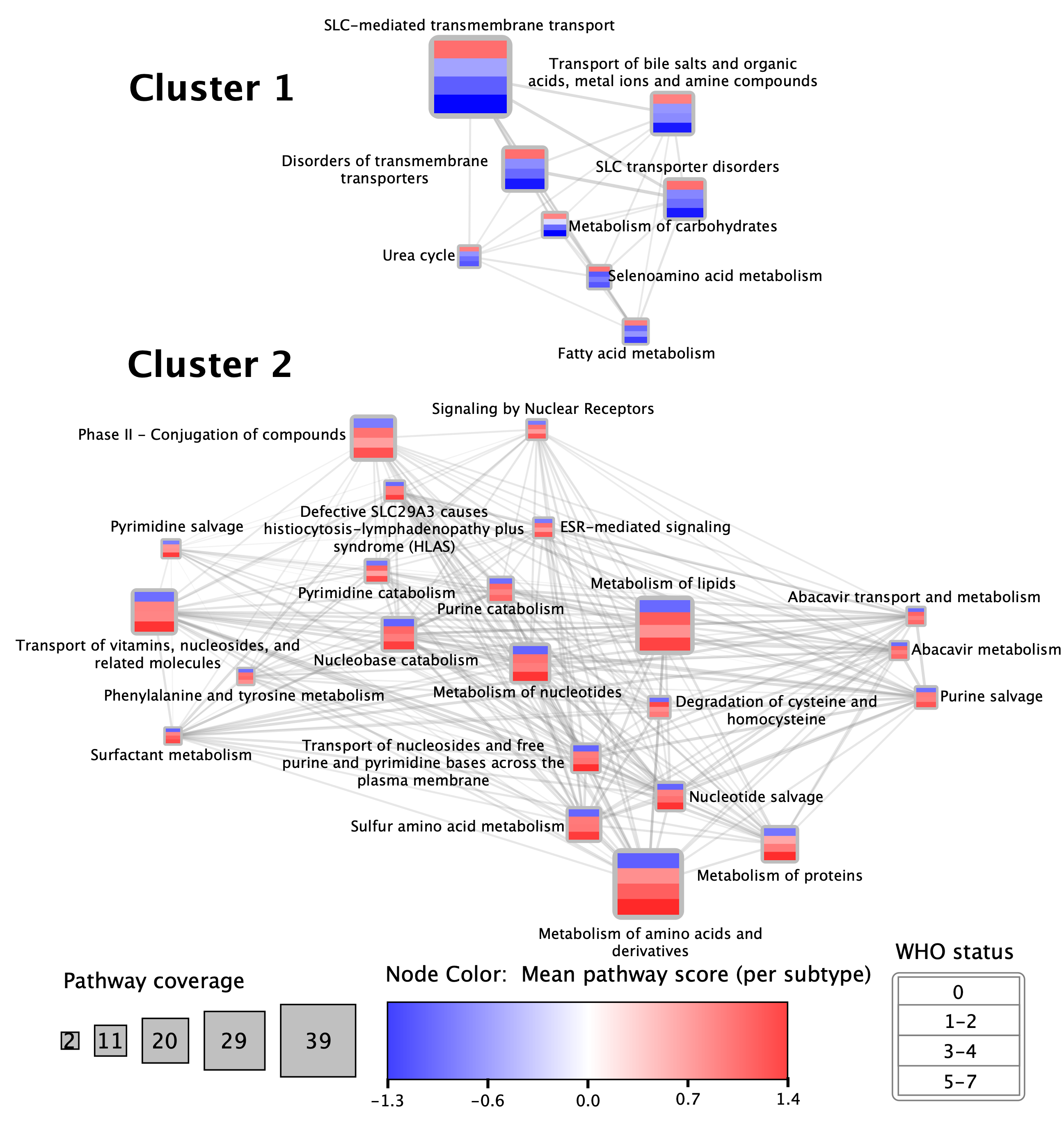


***Fig S9: Pathway clusters derived using hierarchical clustering on COVID dataset transformed to pathway scores using kPCA (top 30 pathways)****. Node fill colour represents mean pathway score across each WHO status level. Edge weight represents Spearman correlation between pathway scores. Only edges with* $\rho$ $\geq$ *0.4 are shown.*

***Table S2: Runtimes of ssPA methods, alongside GSEA* for comparison to conventional PA methods (average across 10 iterations).*** **fGSEA implementation used* [49]

| Method | Mean wall time (seconds) |
| --- | --- |
| SVD | 0.43 |
| ssGSEA | 9.62 |
| GSVA | 3.32 |
| z-score | 0.28 |
| ssClustPA | 9.54 |
| kPCA | 2.10 |
| GSEA | 5.10 |

**Table S3: Over-representation analysis results from IBD dataset.**

| Pathway ID | Hits | P-value | P-adjust | Pathway name |
| --- | --- | --- | --- | --- |
| R-HSA-73884 | 4/4 | 0.06 | 0.985 | Base Excision Repair |
| R-HSA-73929 | 4/4 | 0.06 | 0.985 | Base-Excision Repair, AP Site Formation |
| R-HSA-83936 | 10/14 | 0.082 | 0.985 | Transport of nucleosides and free purine and pyrimidine bases across the plasma membrane |
| R-HSA-15869 | 14/21 | 0.084 | 0.985 | Metabolism of nucleotides |
| R-HSA-6798163 | 3/3 | 0.122 | 0.985 | Choline catabolism |
| R-HSA-74259 | 6/8 | 0.139 | 0.985 | Purine catabolism |
| R-HSA-8956321 | 10/15 | 0.141 | 0.985 | Nucleotide salvage |
| R-HSA-425397 | 16/26 | 0.148 | 0.985 | Transport of vitamins, nucleosides, and related molecules |
| R-HSA-8956319 | 11/17 | 0.155 | 0.985 | Nucleobase catabolism |
| R-HSA-159418 | 4/5 | 0.183 | 0.985 | Recycling of bile acids and salts |
| R-HSA-112310 | 8/12 | 0.185 | 0.985 | Neurotransmitter release cycle |
| R-HSA-74217 | 5/7 | 0.221 | 0.985 | Purine salvage |
| R-HSA-888590 | 2/2 | 0.247 | 0.985 | GABA synthesis, release, reuptake and degradation |
| R-HSA-2046105 | 2/2 | 0.247 | 0.985 | Linoleic acid (LA) metabolism |
| R-HSA-71240 | 2/2 | 0.247 | 0.985 | Tryptophan catabolism |
| R-HSA-2029485 | 2/2 | 0.247 | 0.985 | Role of phospholipids in phagocytosis |
| R-HSA-422085 | 2/2 | 0.247 | 0.985 | Synthesis, secretion, and deacylation of Ghrelin |
| R-HSA-888593 | 2/2 | 0.247 | 0.985 | Reuptake of GABA |
| R-HSA-2142753 | 2/2 | 0.247 | 0.985 | Arachidonic acid metabolism |
| R-HSA-2162123 | 2/2 | 0.247 | 0.985 | Synthesis of Prostaglandins (PG) and Thromboxanes (TX) |
| R-HSA-193807 | 2/2 | 0.247 | 0.985 | Synthesis of bile acids and bile salts via 27-hydroxycholesterol |
| R-HSA-1222556 | 2/2 | 0.247 | 0.985 | ROS and RNS production in phagocytes |
| R-HSA-2029480 | 2/2 | 0.247 | 0.985 | Fcgamma receptor (FCGR) dependent phagocytosis |
| R-HSA-444209 | 8/13 | 0.282 | 0.985 | Free fatty acid receptors |
| R-HSA-2046104 | 3/4 | 0.309 | 0.985 | alpha-linolenic (omega3) and linoleic (omega6) acid metabolism |
| R-HSA-109582 | 3/4 | 0.309 | 0.985 | Hemostasis |
| R-HSA-442660 | 4/6 | 0.339 | 0.985 | Na+/Cl- dependent neurotransmitter transporters |
| R-HSA-73894 | 4/6 | 0.339 | 0.985 | DNA Repair |
| R-HSA-194068 | 4/6 | 0.339 | 0.985 | Bile acid and bile salt metabolism |
| R-HSA-193368 | 4/6 | 0.339 | 0.985 | Synthesis of bile acids and bile salts via 7alpha-hydroxycholesterol |
| R-HSA-192105 | 4/6 | 0.339 | 0.985 | Synthesis of bile acids and bile salts |
| R-HSA-71291 | 19/35 | 0.351 | 0.985 | Metabolism of amino acids and derivatives |
| R-HSA-168249 | 5/8 | 0.358 | 0.985 | Innate Immune System |
| R-HSA-168256 | 5/8 | 0.358 | 0.985 | Immune System |
| R-HSA-2980736 | 8/14 | 0.388 | 0.985 | Peptide hormone metabolism |
| R-HSA-112316 | 8/14 | 0.388 | 0.985 | Neuronal System |
| R-HSA-8978868 | 8/14 | 0.388 | 0.985 | Fatty acid metabolism |
| R-HSA-112315 | 8/14 | 0.388 | 0.985 | Transmission across Chemical Synapses |
| R-HSA-5619102 | 11/20 | 0.402 | 0.985 | SLC transporter disorders |
| R-HSA-373076 | 15/28 | 0.414 | 0.985 | Class A/1 (Rhodopsin-like receptors) |
| R-HSA-425407 | 25/48 | 0.427 | 0.985 | SLC-mediated transmembrane transport |
| R-HSA-556833 | 16/31 | 0.492 | 0.985 | Metabolism of lipids |
| R-HSA-416476 | 10/19 | 0.494 | 0.985 | G alpha (q) signalling events |
| R-HSA-1614635 | 4/7 | 0.496 | 0.985 | Sulfur amino acid metabolism |
| R-HSA-418555 | 4/7 | 0.496 | 0.985 | G alpha (s) signalling events |
| R-HSA-9664433 | 4/7 | 0.496 | 0.985 | Leishmania parasite growth and survival |
| R-HSA-9662851 | 4/7 | 0.496 | 0.985 | Anti-inflammatory response favouring Leishmania parasite infection |
| R-HSA-196807 | 4/7 | 0.496 | 0.985 | Nicotinate metabolism |
| R-HSA-197264 | 4/7 | 0.496 | 0.985 | Nicotinamide salvaging |
| R-HSA-5619108 | 4/7 | 0.496 | 0.985 | Defective SLC27A4 causes ichthyosis prematurity syndrome (IPS) |
| R-HSA-9658195 | 4/7 | 0.496 | 0.985 | Leishmania infection |
| R-HSA-804914 | 4/7 | 0.496 | 0.985 | Transport of fatty acids |
| R-HSA-73614 | 4/7 | 0.496 | 0.985 | Pyrimidine salvage |
| R-HSA-9660821 | 4/7 | 0.496 | 0.985 | ADORA2B mediated anti-inflammatory cytokines production |
| R-HSA-73621 | 5/9 | 0.496 | 0.985 | Pyrimidine catabolism |
| R-HSA-390696 | 2/3 | 0.497 | 0.985 | Adrenoceptors |
| R-HSA-70635 | 2/3 | 0.497 | 0.985 | Urea cycle |
| R-HSA-6806667 | 2/3 | 0.497 | 0.985 | Metabolism of fat-soluble vitamins |
| R-HSA-6814848 | 2/3 | 0.497 | 0.985 | Glycerophospholipid catabolism |
| R-HSA-76002 | 2/3 | 0.497 | 0.985 | Platelet activation, signaling and aggregation |
| R-HSA-8963693 | 2/3 | 0.497 | 0.985 | Aspartate and asparagine metabolism |
| R-HSA-9018678 | 2/3 | 0.497 | 0.985 | Biosynthesis of specialized proresolving mediators (SPMs) |

**Table S4: Top 50 features in random forest model based on IBD data.** Features are ranked by mean AUC decrease which is computed by permuting each of the features individually.

| Pathway ID | Mean AUC decrease | Pathway name |
| --- | --- | --- |
| R-HSA-3296197 | 0.00979 | Hydroxycarboxylic acid-binding receptors |
| R-HSA-196854 | 0.00391 | Metabolism of vitamins and cofactors |
| R-HSA-5652084 | 0.00333 | Fructose metabolism |
| R-HSA-9018683 | 0.00263 | Biosynthesis of DPA-derived SPMs |
| R-HSA-428643 | 0.00262 | Organic anion transporters |
| R-HSA-9658195 | 0.00194 | Leishmania infection |
| R-HSA-211935 | 0.00172 | Fatty acids |
| R-HSA-9018678 | 0.00166 | Biosynthesis of specialized proresolving mediators (SPMs) |
| R-HSA-177128 | 0.00148 | Conjugation of salicylate with glycine |
| R-HSA-1614558 | 0.00147 | Degradation of cysteine and homocysteine |
| R-HSA-156587 | 0.00146 | Amino Acid conjugation |
| R-HSA-156581 | 0.00135 | Methylation |
| R-HSA-192105 | 0.00126 | Synthesis of bile acids and bile salts |
| R-HSA-159424 | 0.00119 | Conjugation of carboxylic acids |
| R-HSA-193368 | 0.00117 | Synthesis of bile acids and bile salts via 7alpha-hydroxycholesterol |
| R-HSA-427601 | 0.00112 | Multifunctional anion exchangers |
| R-HSA-197264 | 0.00104 | Nicotinamide salvaging |
| R-HSA-75105 | 0.00104 | Fatty acyl-CoA biosynthesis |
| R-HSA-418555 | 0.001 | G alpha (s) signalling events |
| R-HSA-2029485 | 0.00094 | Role of phospholipids in phagocytosis |
| R-HSA-194068 | 0.00084 | Bile acid and bile salt metabolism |
| R-HSA-3134975 | 0.00084 | Regulation of innate immune responses to cytosolic DNA |
| R-HSA-83936 | 0.00084 | Transport of nucleosides and free purine and pyrimidine bases across the plasma membrane |
| R-HSA-209776 | 0.00081 | Metabolism of amine-derived hormones |
| R-HSA-2046105 | 0.0008 | Linoleic acid (LA) metabolism |
| R-HSA-1369062 | 0.00078 | ABC transporters in lipid homeostasis |
| R-HSA-9025106 | 0.00077 | Biosynthesis of DPAn-6 SPMs |
| R-HSA-211859 | 0.00075 | Biological oxidations |
| R-HSA-351143 | 0.00075 | Agmatine biosynthesis |
| R-HSA-425397 | 0.00075 | Transport of vitamins, nucleosides, and related molecules |
| R-HSA-9711123 | 0.00074 | Cellular response to chemical stress |
| R-HSA-9660821 | 0.00072 | ADORA2B mediated anti-inflammatory cytokines production |
| R-HSA-196849 | 0.00072 | Metabolism of water-soluble vitamins and cofactors |
| R-HSA-156580 | 0.0007 | Phase II - Conjugation of compounds |
| R-HSA-390696 | 0.0007 | Adrenoceptors |
| R-HSA-211897 | 0.00067 | Cytochrome P450 - arranged by substrate type |
| R-HSA-189483 | 0.00065 | Heme degradation |
| R-HSA-162582 | 0.00065 | Signal Transduction |
| R-HSA-156584 | 0.00064 | Cytosolic sulfonation of small molecules |
| R-HSA-418346 | 0.00063 | Platelet homeostasis |
| R-HSA-9006934 | 0.00062 | Signaling by Receptor Tyrosine Kinases |
| R-HSA-5218920 | 0.00061 | VEGFR2 mediated vascular permeability |
| R-HSA-420499 | 0.0006 | Class C/3 (Metabotropic glutamate/pheromone receptors) |
| R-HSA-8963743 | 0.00058 | Digestion and absorption |
| R-HSA-9707564 | 0.00058 | Cytoprotection by HMOX1 |
| R-HSA-70921 | 0.00057 | Histidine catabolism |
| R-HSA-15869 | 0.00057 | Metabolism of nucleotides |
| R-HSA-74259 | 0.00056 | Purine catabolism |
| R-HSA-5619102 | 0.00055 | SLC transporter disorders |
| R-HSA-211981 | 0.00054 | Xenobiotics |
